# Improved metabolomic data-based prediction of depressive symptoms using nonlinear machine learning with feature selection

**DOI:** 10.1038/s41398-020-0831-9

**Published:** 2020-05-19

**Authors:** Yuta Takahashi, Masao Ueki, Makoto Yamada, Gen Tamiya, Ikuko N. Motoike, Daisuke Saigusa, Miyuki Sakurai, Fuji Nagami, Soichi Ogishima, Seizo Koshiba, Kengo Kinoshita, Masayuki Yamamoto, Hiroaki Tomita

**Affiliations:** 1 Graduate School of Medicine, Tohoku University, Sendai, Japan; 2 Tohoku Medical Megabank Organization, Tohoku University, Sendai, Japan; 3 International Research Institute of Disaster Science, Tohoku University, Sendai, Japan; 4 RIKEN Center for Advanced Intelligence Project, Tokyo, Japan; 5 Graduate School of Information Sciences, Tohoku University, Sendai, Japan; 6 Institute for Development Aging and Cancer, Tohoku University, Sendai, Japan

**Keywords:** Personalized medicine, Diagnostic markers

## Abstract

To solve major limitations in algorithms for the metabolite-based prediction of psychiatric phenotypes, a novel prediction model for depressive symptoms based on nonlinear feature selection machine learning, the Hilbert–Schmidt independence criterion least absolute shrinkage and selection operator (HSIC Lasso) algorithm, was developed and applied to a metabolomic dataset with the largest sample size to date. In total, 897 population-based subjects were recruited from the communities affected by the Great East Japan Earthquake; 306 metabolite features (37 metabolites identified by nuclear magnetic resonance measurements and 269 characterized metabolites based on the intensities from mass spectrometry) were utilized to build prediction models for depressive symptoms as evaluated by the Center for Epidemiologic Studies-Depression Scale (CES-D). The nested fivefold cross-validation was used for developing and evaluating the prediction models. The HSIC Lasso-based prediction model showed better predictive power than the other prediction models, including Lasso, support vector machine, partial least squares, random forest, and neural network. l-leucine, 3-hydroxyisobutyrate, and gamma-linolenyl carnitine frequently contributed to the prediction. We have demonstrated that the HSIC Lasso-based prediction model integrating nonlinear feature selection showed improved predictive power for depressive symptoms based on metabolome data as well as on risk metabolites based on nonlinear statistics in the Japanese population. Further studies should use HSIC Lasso-based prediction models with different ethnicities to investigate the generality of each risk metabolite for predicting depressive symptoms.

## Introduction

Metabolomics, an emerging field involving the measurement of a comprehensive small-molecule profile in a biological sample in a single experiment, may be one of the most promising approaches for providing global insight into the mechanisms underlying depressive symptoms^1^. A profile of metabolites in plasma, which are accessed relatively easily in clinical situations, can be an intermediate phenotype between the genome/transcriptome and general conditions of the body reflecting phenomena relevant to depression. There is increasing evidence indicating the potential contribution of plasma metabolome profiles to the understanding of depression^1–3^.

Instead of utilizing a single metabolite as a risk factor to predict depression, prediction models utilizing metabolomic data have recently been proposed to improve the accuracy of the prediction of depressive symptoms^4,5^. However, these initial trials to identify specific metabolite markers have not been well replicated^2,3,6^. As major limitations underlying current attempts to detect the risk metabolites for depression, issues regarding statistics and sample size remain to be addressed.

Among the statistical challenges in dealing with metabolomic data, the abundance of data encompasses both useful and useless information for prediction, and nonlinear characteristics of data may be major problems to be solved. Comprehensive metabolomic data include a large number (several hundreds) of variables not contributing to prediction accuracy, consisting of variables not associated with the phenotype of interest, and variables highly correlated with other variables without adding new information to the prediction model, which are referred to as redundancies. In addition to the fact that the prediction model utilizing multiple linear/logistic regression cannot be built with a larger number of metabolites than samples, prediction models including useless predictor variables result in insufficient prediction accuracy due to multicollinearity or overfitting. One of the effective ways to avoid multicollinearity or overfitting is to choose a set of prioritized predictor variables, a process referred to as feature selection. Machine learning is frequently utilized for this purpose because it is difficult to select the best set of predictive variables to increase prediction accuracy based on a list of statistics calculated for each metabolite based on traditional statistics^7^.

Least absolute shrinkage and selection operator (Lasso) is one of the most commonly used machine learning prediction methods incorporating feature selection^8^. Although Lasso solves the first limitation, the abundance of metabolomic data, by applying feature selection, it does not handle the second limitation, the nonlinearity of metabolomic data, because it stands on the assumption that all variables have linear relationships. Nonlinear associations were reported among metabolites^9^, between metabolite concentrations and covariates, e.g., age^10^ and body mass index (BMI)^11^, and between depressive phenotype and covariates^12^. One of the potential solutions addressing nonlinearity among metabolites is the implementation of kernel-based machine learning methods, such as a support vector machine (SVM) for categorical phenotypes and kernel regression (KR) for quantitative phenotypes; these are the most commonly utilized machine learning methods to handle data with nonlinear relationships among variables^7^. However, these methods can be affected by overfitting in certain situations using omics datasets. The omics datasets can be characterized by a small sample size, a large number of features, and only a small proportion of useful features for prediction. Although SVM uses support vectors to protect the methods against overfitting to a certain degree, several previous studies have reported that SVM, which does not integrate a feature selection process, is more overfitted than other models with feature selection based on omics datasets^13,14^.

Hilbert–Schmidt independence criterion (HSIC) Lasso is a novel nonlinear feature selection model developed by Yamada et al.^15^ to overcome the above limitations. HSIC Lasso extracts a set of predictor variables, which are dependent on a response variable and independent from other selected predictor variables, where dependencies between variables are evaluated by HSIC statistics, which is a nonparametric score for dependency^15,16^. Unlike Lasso, which incorporates both feature selection and prediction algorithms, HSIC Lasso is solely used for feature selection; therefore, it should be combined with other algorithms for prediction. Herein, we propose a novel statistical approach combining HSIC Lasso-based feature selection and SVM- or KR-based prediction to predict a depressive phenotype based on metabolomic data.

In addition to the statistical issues, another major limitation is that most previous studies have had limited sample sizes to perform machine learning^3,4,6^. Although machine learning is an effective approach for analyzing metabolomic data, it usually requires larger sample sizes than traditional statistics because of the much greater number of degrees of freedom that ought to be covered^17^. However, the sample sizes of the prior metabolomic studies to predict depressive symptoms, utilizing either traditional statistics or machine learning, included up to a couple of hundred samples^3,4,6^.

In this study, we applied the novel nonparametric prediction model utilizing HSIC Lasso and SVM/KR to the prediction of depressive symptoms based on metabolomic data from 897 plasma samples, which is more than double the number of samples in the largest previous studies^3,4,6^. The samples were collected by the Tohoku University Tohoku Medical Megabank Organization to survey the health condition of residents of prefectures that were primarily affected by the Great East Japan Earthquake and Tsunami^18^. The predictive powers of HSIC Lasso-based prediction models were compared with those of state-of-the-art prediction models, including Lasso, SVM/KR without feature selection, random forest, partial least squares (PLS), sparse PLS (SPLS), neural network, and multiple linear/logistic regression. The metabolites extracted by feature selection using our model were also investigated by comparing them with the metabolites selected by other models or with metabolites with small *P* values in the traditional statistical analyses.

## Materials and methods

### Study population

This study has a population-based cross-sectional design. The subjects included in the first batch (*n* = 1008) of the Japanese Multi Omics Reference Panel (jMorp)^19^ were included in the current analyses. There were 48 subjects (4.7%) whose CES-D scores were missing (listwise deletion) and 63 subjects (6.2%) whose CES-D answers were unreliable under the criteria described below. After the exclusion of these 111 subjects, 897 subjects were subjected to the analyses described below. All protocols of the studies were approved by the Ethics Committee of Tohoku University. Written informed consent was obtained from all subjects at the time of study enrolment.

### Outcome measures

The Center for Epidemiologic Studies-Depression Scale (CES-D) was used as an indicator of depressive symptoms^20^. The details of the CES-D scores are provided in the Supplementary Methods. As outcome measures, not only quantitative CES-D scores but also binary CES-D traits using cutoff values were utilized. We set two cutoff values (≥16 and ≥19 to define the depressive group) for prediction analyses for the binary CES-D traits and compared the results.

### Nuclear magnetic resonance (NMR) measurements and mass spectrometry (MS) measurements

NMR and MS measurements are detailed in Supplementary Methods. In brief, plasma was prepared and stored at −80 °C. Metabolites were extracted using a standard methanol extraction procedure. All NMR experiments were performed at 298 K using a Bruker Advance 600 MHz spectrometer (Bruker BioSpin, Billerica, MA, USA). After standard 1D nuclear Overhauser effect spectroscopy (NOESY) and Carr–Purcell–Meiboom–Gill (CPMG) spectra were measured for each sample, data were processed utilizing the Chenomx NMR Suite (Chenomx, Edmonton, Canada). Identification and quantification of metabolites were performed using the target profiling approach implemented in the Chenomx Profiler module.

Ultrahigh-performance liquid chromatography quadrupole time-of-flight MS analysis was performed on an ACQUITY Ultra Performance liquid chromatography I-class system (Waters Corp., Milford, MA, USA), which was interfaced with a Waters Synapt G2-Si quadrupole time-of-flight MS with an electrospray ionization (ESI) system utilized in positive-ion mode. A C18 column (ACQUITY HSS T3, Waters Corp.) was used for liquid chromatography separation. The data collection were performed using MassLynx, v4.1 software (Waters Corp.). A NANOSPACE SI-2 HPLC (Shiseido, Tokyo, Japan) and a Q Exactive Orbitrap MS (Thermo Fisher Scientific, Waltham, MA, USA) equipped with a heated-ESI-II source were integrated into the liquid chromatography Fourier Transform MS system for negative ion mode. A HILIC column (ZIC-pHILIC, SeQuant, Darmstadt, Germany) was used for liquid chromatography separation. The data collection was performed using Xcalibur v4.1 software (Thermo Fisher Scientific).

### Covariates

Sex, age, BMI, marital status, damage from the Great East Japan Earthquake, antidepressant intake, Lubben Social Network Scale 6, and social capital score were utilized as covariates. These covariates, as well as metabolites, were included in the variable selection, and selected covariates in each fold of the outer-loop cross-validation are shown in Supplementary Table S1.

Sex, age, BMI, and antidepressant intake were frequently used as covariates in previous studies^2,3,6^. Experiencing the natural disaster, which was associated with the CES-D score^21,22^, was also reported to be associated with metabolite profile (i.e., lipids, blood sugar, and inflammation-related factors)^23^, which is possibly due to environmental change (changing residence or living in a shelter) and chronic stress. The damage from the Great East Japan Earthquake was coded based on the categories of house damage evaluated by the local government following the National Damage Certification Standards of Disaster as follows: 4 = entirely collapsed (uninhabitable), 3 = large-scale damage (requires significant repairs), 2 = half-scale damage (habitable with repairs), 1 = small-scale damage, and 0 = no damage^21^.

In addition, there is accumulating evidence that environmental factors related to lifestyle (i.e., marital status and social engagement), which are associated with depressive symptoms, are also associated with metabolite profiles (especially lipids)^24–27^ through dietary and other health behaviors^28,29^. Social engagement in particular is one of the strongest environmental factors for depressive symptoms in the Japanese population suffering from the natural disaster^22^. The questionnaire-based scales that have already been validated in Japanese people (i.e., the Lubben Social Network Scale 6^30^ and the social capital score^22^) were utilized to evaluate social engagement. Since we are interested in the association between metabolites and CES-D score adjusted by environmental factors, these environmental factors were included as covariates.

### Cross-validation

Evaluation of the predictive powers and tuning parameters for each prediction model was based on fivefold cross-validation. In fivefold cross-validation, all the subjects were randomly split into five roughly equal-sized groups. We predicted each group (test dataset) using the model fitted on the remaining four groups of data as training datasets. We repeated this process for each of the five groups and obtained five estimates of predictive power. This cross-validation for evaluating predictive power was referred to as the “outer loop”. Predictive power was evaluated based on the predictive correlation coefficient (i.e., Pearson’s correlation coefficient between the predicted and measured CES-D scores) for quantitative traits and the area under the curve (AUC) for binary traits.

The parameters for the prediction models (i.e., hyperparameters) were also selected based on fivefold cross-validation. Training datasets from the outer loop were split into five parts again, and each group was used as a test dataset; the remaining four parts were used as training datasets repeatedly. The mean of the estimates of the five predictive powers were calculated, and the parameters that gave the maximum predictive power were used as the optimized parameters. This cross-validation is also known as the “first inner loop”. If the prediction models consisted of separate algorithms for feature selection and prediction, the parameters for the feature selection were tuned in the first inner loop; then, the training datasets from the first inner loop were further split into five datasets, and fivefold cross-validation was performed yet again to select the optimized parameters for the prediction part (the “second inner loop”). The subject sets included in the outer loop, the first inner loop, and the second inner loop were common in all the prediction models.

### HSIC Lasso-based prediction model

HSIC Lasso was used for feature selection. Although “Lasso” is included in the method name, HSIC Lasso can be categorized as a screening method, such as sure independence screening (SIS)^31^, rather than as a prediction method, since HSIC Lasso transforms the output variable y (e.g., CES-D score) to a Gram matrix L and cannot directly estimate y^15^. SIS is a widely studied feature selection method in the statistics community^32^; it first screens a small number of features without considering the prediction accuracy of the constructed prediction model (e.g., using mutual information and correlation) and then predicts the output by using an existing prediction model with the screened features. HSIC Lasso can screen features by considering feature–feature nonlinear relationships in addition to feature–output relationships, unlike SIS, which only considers the feature and output relation to screen features. HSIC Lasso’s approach for feature selection is similar to the minimal redundancy maximal relevance criterion^33^, which selects the features that were not redundant (independent) with each other and had maximal statistical dependency on the output variable. Similar to classical Lasso-based feature selection, the minimal redundancy maximal relevance criterion focuses on mutual information, does not consider the interaction effects between features and can be performed for datasets whose sample sizes are smaller than the feature size (*n* < *p*). In contrast to classical Lasso, HSIC Lasso uses HSIC statistics, a kernel-based independence measure, to assess the nonlinear dependency between two variables (i.e., between two features or between the output variable and a feature). HSIC statistics takes a nonnegative value and is zero only if the two variables are statistically independent.

After selecting features with HSIC Lasso, we built a model for predicting quantitative response variables via KR and for predicting binary response variables via SVM using the R kernlab and CVST packages. The characteristics of this new model, along with those of the frequently utilized conventional models, are summarized in Table 1. For KR and SVM, a radial basis function kernel was utilized. For the tuning parameters, the first inner loop of the fivefold cross-validation was used to select the optimal number of features selected by HSIC Lasso. The second inner loop of the fivefold cross-validation was used to select the optimal sigma and lambda/C parameters for KR/SVM.

**Table 1:** Model characteristics.

	Our model (HSIC Lasso + SVM/KR)	HSIC Lasso	Lasso	SVM/KR	Random forest	Partial least squares	Sparse partial least squares	Multiple linear/logistic regression
Prediction	**Performed**	Not performed	**Performed**	**Performed**	**Performed**	**Performed**	**Performed**	**Performed**
Feature selection	**Performed**	**Performed**	**Performed**	Not performed	Not performed	Not performed	**Performed**	Not performed
Nonlinear association between variables	**Detectable**	**Detectable**	Not detectable	**Detectable**	**Detectable**	Not detectable	Not detectable	Not detectable

Notes: Preferable properties for handling metabolomic data are in bold.

*HSIC* Lasso Hilbert–Schmidt Independence Criterion Lasso, *SVM* support vector machine, *KR* kernel regression.

### Additional prediction models for comparison to the HSIC Lasso-based prediction model

Lasso was included as a feature selection and prediction model with linear assumptions using the R glmnet package. The tuning parameter for Lasso (lambda) was determined by the first inner loop of fivefold cross-validation. SVM/KR was utilized as a prediction model without linear assumptions. In addition to the SVM/KR without feature selection, SVM/KR with *P* < 0.05 variables, SVM/KR with Lasso-based feature selection, and SVM/KR with only covariates were performed. For SVM/KR without feature selection, SVM/KR with *P* < 0.05 variables, and SVM/KR using only covariates, the optimal sigma and lambda/C parameters were selected in the first inner loop of the fivefold cross validation. For SVM/KR with Lasso-based feature selection, the optimal parameters for Lasso (lambda) and the optimal parameters for SVM/KR were selected in the first and second inner loops of the cross-validation, respectively. Random forest is a nonlinear machine learning method without feature selection, which builds multiple decision trees and merges them together to obtain a more accurate and stable prediction^34^. Using the R randomForest package, the mtry parameter (the number of variables randomly sampled as candidates at each split) was optimized with respect to the Out-of-Bag error estimate. The number of trees in the random forest was set to 500, which is the default value in the R randomForest package. The prediction power of various numbers of trees in the random forests is shown in Supplementary Table S2. PLS is a technique that reduces the predictors to a smaller set of uncorrelated components and performs least squares regression/classification on these components instead of on the original data^7^. SPLS is a modified PLS that directly imposes sparsity on the dimension reduction step of PLS to achieve accurate prediction and variable selection^35^. For PLS, the number of components was decided by the first inner loop of the fivefold cross-validation. For SPLS, the number of components and the thresholding parameter (eta) were decided by the first inner loop of the fivefold cross-validation. The R caret package was utilized for PLS and SPLS. A neural network, which is inspired by biological neural networks, is a computational model whose architecture is composed of layers and nodes^36^. A neural network can be a nonlinear prediction model when a nonlinear activation function is used. For the neural network, two hidden layers with 128 nodes and the Rectified Linear Unit activation function were utilized. This architecture was determined based on previous studies^37,38^ and preliminary trials. The epoch size for training was selected based on the first inner loop of the fivefold cross-validation. The Python keras package was utilized. The prediction power of various neural network architectures is shown in Supplementary Table S3.

## Results

### Demographic information

The demographics of the subjects between the high and low CES-D groups using a cutoff of 16 are shown in Table 2, and those using a cutoff of 19 are shown in Supplementary Table S4. The percentage of females, marital status, house damage from the 2011 Great East Japan Earthquake and Tsunami, medications, and social engagement scores were significantly different between the high and low CES-D groups. The self-reported posttraumatic stress disorder (PTSD) symptoms are also shown in Table 2 and Supplementary Table S4. The proportion of subjects who complained of PTSD symptoms was significantly higher in the high CES-D group.

**Table 2:** Demographic information.

	High CES-D	Low CES-D	*P* value^a^
CES-D range	≥16	≤15	
CES-D, mean (SD)	22.2 (6.4)	9.8 (3.8)	1.26 × 10^−106^
Subjects	298	599	
Percentage of females	64.0%	54.5%	7.87 × 10^−3^
Age, mean (SD)	56.8 (11.7)	58.2 (11.6)	0.105
BMI, mean (SD)	23.50 (4.13)	23.49 (3.31)	0.972
*Marital status*			
Married	226 (75.83%)	510 (85.14%)	7.28 × 10^−3^
Widowed	26 (8.72%)	33 (5.50%)	
Divorced	18 (6.04%)	19 (3.17%)	
Single	28 (9.39%)	37 (6.17%)	
*House damage from the GEJE*			
Total collapse	75 (25.2%)	75 (12.5%)	8.80 × 10^−5^
Large-scale damage	36 (12.1%)	74 (12.3%)	
Half-scale damage	38 (12.8%)	82 (13.6%)	
Small-scale damage	99 (33.2%)	239 (39.8%)	
No damage	50 (16.8%)	129 (21.5%)	
Medication			
Antidepressants	9 (3.0%)	0 (0.0%)	4.54 × 10^−5^
Hypnotics	57 (19.1%)	18 (3.0%)	2.43 × 10^−15^
Anxiolytics	94 (31.5%)	21 (3.5%)	1.22 × 10^−30^
*Social engagement*			
LSNS-6 score, mean (SD)	14.0 (5.8)	16.23 (5.4)	2.30 × 10^−8^
Social capital score, mean (SD)	5.7 (2.9)	4.44 (2.4)	5.27 × 10^−12^
Gap time between the GEJE and measurement of CES-D (months), mean (SD)	27.3 (1.0)	27.4 (1.0)	0.111
*Self-reported PTSD symptoms*^b^			
1. Intrusive images or nightmares	102 (34.2%)	49 (8.1%)	1.91 × 10^−21^
2. Emotionally upset when reminded of the GEJE	102 (34.2%)	50 (8.3%)	5.63 × 10^−21^
3. Physiological reactions when reminded of the GEJE	45 (15.1%)	16 (2.6%)	2.84 × 10^−11^
4. Avoidance of reminders associated with the GEJE	86 (28.8%)	67 (11.1%)	1.32 × 10^−10^
5. Interference with everyday life	36 (12.0%)	8 (1.3%)	1.41 × 10^−11^

*CES-D* Center for Epidemiologic Studies—Depression Scale, *SD* standard deviation, BMI body mass index, *PTSD* posttraumatic stress disorder, *GEJE* the 2011 Great East Japan Earthquake and Tsunami.

^a^*P* values were calculated using Student’s *t* tests for CES-D, age, BMI, LSNS-6 score, social capital score, and the gap time between the 2011 Great East Japan Earthquake and the CES-D measurement. *P* values were calculated using Fisher’s exact tests for the percentage of females, marital status, house damage from the 2011 Great East Japan Earthquake and Tsunami, medication, and self-reported PTSD symptoms.

^b^Self-reported PTSD symptoms show the number of subjects who answered “Yes” to the following questions in the questionnaire. “Below is a list of problems that people sometimes have after experiencing a traumatic event. Have you experienced the following problems two times or more within one week? 1. Unwanted upsetting memories about the GEJE or bad dreams or nightmares related to the GEJE. 2. Feeling very emotionally upset when reminded of the GEJE. 3. Having physical reactions when reminded of the GEJE (for example, sweating or heart racing). 4. Trying to avoid thoughts or feelings related to the GEJE or trying to avoid activities, situations, or places that remind you of the GEJE or that feel more dangerous since the GEJE. 5. The difficulties have been interfering with your everyday life.” These questions were based on the report by Itoh et al.^46^, which validated a new short version of the Posttraumatic Diagnostic Scale^47^ among Japanese people.

### Predictive powers

**Fig. 1:**
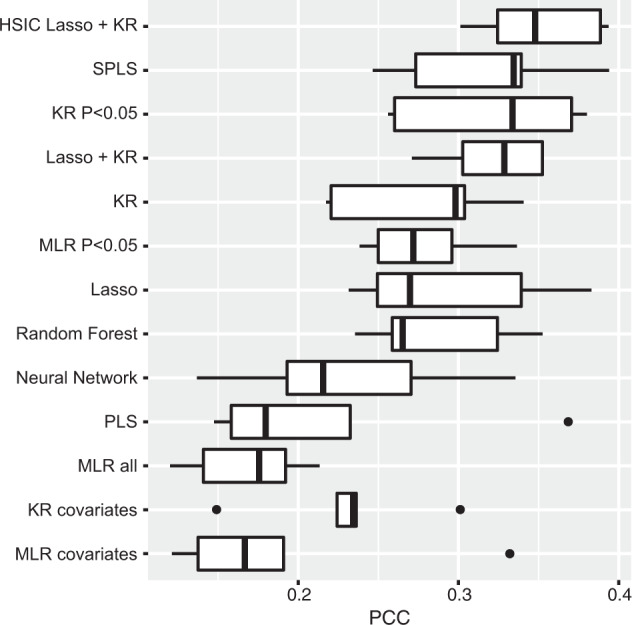
Predictive power for quantitative CES-D scores. Boxplots show the predictive powers in the fivefold cross-validations of each prediction model utilizing CES-D scores as response variables and metabolites and other covariates as predictive variables. Abbreviations: CES-D Center for Epidemiologic Studies-Depression Scale, HSIC Hilbert–Schmidt independence criterion, Lasso least absolute shrinkage and selection operator, KR kernel regression, SPLS sparse partial least squares, KR *P* < 0.05 kernel regression with *P* < 0.05 variables, Lasso + KR kernel regression with variables selected by Lasso, MLR *P* < 0.05 multiple linear regression with *P* < 0.05 variables, PLS partial least squares, MLR all multiple linear regression with all variables, KR covariates kernel regression with only covariates, MLR covariates multiple linear regression with only covariates, PCC predictive correlation coefficient.

**Fig. 2:**
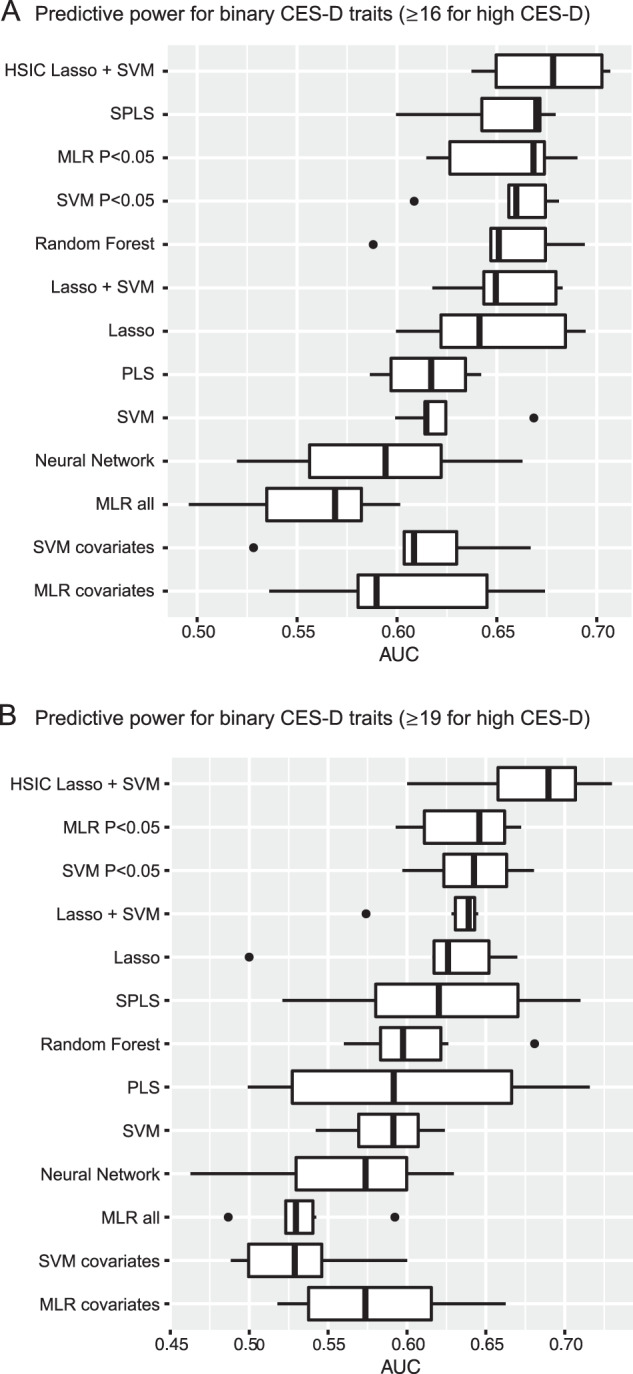
Predictive power for binary CES-D traits. Boxplots show the predictive power in the fivefold cross-validations of each prediction model utilizing binary CES-D traits as response variables and metabolites and other covariates as predictive variables. Abbreviations: CES-D Center for Epidemiologic Studies-Depression Scale, HSIC Hilbert–Schmidt independence criterion, Lasso least absolute shrinkage and selection operator, SVM support vector machine, SPLS sparse partial least squares, MLR *P* < 0.05 multiple logistic regression with *P* < 0.05 variables, SVM *P* < 0.05 support vector machine with *P* < 0.05 variables, Lasso + SVM support vector machine with variables selected by Lasso, PLS partial least squares, MLR all multiple linear regression with all variables, SVM covariates support vector machine with only covariates, MLR covariates multiple logistic regression with only covariates, AUC area under the curve.

The predictive powers of all conducted models are shown in Figs. 1 and 2. The receiver operating characteristic curve for the score that predicts binary traits is shown in Supplementary Fig. S1. In the prediction of both the quantitative CES-D score and the binary CES-D traits, the predictive power of HSIC Lasso with SVM/KR was higher than that of the other prediction models.

### Selected metabolites

During the fivefold cross-validation in the predictions integrating feature selections, five sets of metabolites were selected from the training data (four-fifths of total subjects) to be subjected to validation utilizing the test data (the remaining one fifth). Frequently selected metabolites among the five replications of feature selections can be interpreted as useful metabolites to predict depression. The metabolites with frequencies of more than four times out of the five replicated feature selections for both CES-D quantitative variable models and binary variable models are shown in Table 3. Three metabolites—3-hydroxyisobutyrate (NMR), gamma-linolenyl carnitine (MS in C18 mode) and l-leucine (MS in C18 mode)—were frequently selected in both nonlinear and linear feature selection models. Uric acid (MS in C18 mode) was frequently selected only in linear feature selection models (Lasso and multiple linear/logistic regression with *P* < 0.05), and l-gamma-glutamyl l-leucine (MS in C18 mode) was frequently selected only in multiple linear/logistic regression with *P* < 0.05 (Table 3).

**Table 3:** Frequently selected metabolites in feature selection models by fivefold cross-validation and their *P* values and regression coefficients in multiple regression analyses.

	CES-D score					Binary CES-D traits				
	HSIC Lasso + KR	Lasso	Multiple linear regression <0.05	*P* value^a^	RC^a^	HSIC Lasso + SVM	Lasso	Multiple logistic regression <0.05	*P* value^a^	RC^a^
Number of selected metabolites for prediction (mean ± standard deviation)	9.6 ± 2.8	13.4 ± 6.4	16.0 ± 4.0	NA	NA	9.4 ± 6.2	23.4 ± 6.7	26.4 ± 8.0	NA	NA
*Metabolites selected in both nonlinear and linear models*										
3-Hydroxyisobutyrate	**5/5**	**5/5**	**5/5**	6.67 × 10^−4^	−0.832	**5/5**	**5/5**	**5/5**	1.78 × 10^−4^	−0.320
NMR										
Gamma-linolenyl carnitine	**4/5**	**5/5**	**5/5**	1.44 × 10^−3^	−0.758	**5/5**	**5/5**	**5/5**	5.20 × 10^−3^	−0.243
MS C18										
l-leucine	**4/5**	3/5	**4/5**	3.52 × 10^−3^	−0.753	**5/5**	**5/5**	**5/5**	1.06 × 10^−3^	−0.286
MS C18										
*Metabolites selected only in linear models*										
Uric acid	0/5	**5/5**	**5/5**	3.03 × 10^−4^	−1.024	0/5	**5/5**	**4/5**	9.61 × 10^−3^	−0.239
MS C18										
*Metabolites selected only by P values from multiple regression*										
l-gamma-glutamyl-l-leucine^b^	1/5	1/5	**5/5**	9.19 × 10^−3^	−0.679	0/5	0/5	**5/5**	1.82 × 10^−2^	−0.206
MS C18										

Abbreviations: *CES-D* Center for Epidemiologic Studies-Depression Scale, *HSIC Lasso* Hilbert–Schmidt independence criterion lasso, *KR* kernel regression, *RC* regression coefficients, *SVM* support vector machine, *NMR* nuclear magnetic resonance spectroscopy, *MS C18* mass spectrometry in C18 mode.

The frequencies are shown as *n*/5, which means that the metabolites were utilized for prediction *n* times out of five replicated feature selections.

^a^*P* value and regression coefficients are adjusted by sex, age, body mass index, marital status, the degree of damage from the Great East Japan Earthquake, antidepressant use, Lubben Social Network Scale 6, and social capital scale in multiple linear/logistic regression.

^b^In the jMorp metabolomic database, l-gamma-glutamyl-l-leucine and l-gamma-glutamyl-l-isoleucine were not differentiated for one of the features selected by *P* values from multiple regression, and standard reagents, i.e., H-Glu(Leu-OH)-OH (BACHEM, Budendorf, Switzerland) and l-gamma-glutamyl-l-isoleucine (Santa Cruz Biotechnology, Heidelberg, Germany), were utilized to determine l-gamma-glutamyl-l-leucine as the detected metabolite for the feature in the current study.

### Dependencies among CES-D and features

Dependencies among CES-D score, metabolites, and covariates were investigated utilizing HSIC statistics. The HSIC statistics between CES-D score, covariates, and the five selected metabolites are shown in Table 4. HSIC statistics between all possible variable pairs (CES-D score, covariates, and all metabolites) are shown in Supplementary Table S5.

**Table 4:** Dependencies among CES-D score, metabolites, and covariates based on HSIC statistics.

	Response variable	Metabolites selected in both nonlinear and linear models			Metabolites selected only in linear models	Metabolite selected only by *P* values from multiple regression
	CES-D score	3-Hydroxyisobutyrate	Gamma-linolenyl carnitine	l-Leucine	Uric acid	l-gamma-glutamyl-l-leucine
		NMR	MS C18	MS C18	MS C18	MS C18
*Covariates*						
Sex	0.9	5.6	0.7	15.7	29.5	15.8
Age	1.0	1.3	1.5	1.5	1.7	3.0
BMI	1.0	2.3	0.3	3.6	4.7	2.8
Marital status	0.6	0.1	0.5	0.2	0.1	0.3
Damage from the Great East Japan Earthquake	0.9	0.5	0.1	0.6	0.5	0.5
Antidepressants	0.4	0.3	0.1	0.2	0.0	0.1
LSNS-6	1.6	0.2	0.2	0.3	0.5	0.1
Social capital	1.4	0.6	0.4	0.3	0.2	0.2
sum	7.2	11.1	4.1	22.7	37.6	23.2
*Metabolites*						
3-Hydroxyisobutyrate						
NMR	1.2	NA	0.6	6.9	2.7	5.7
Gamma-linolenyl carnitine						
MS C18	0.9	0.6	NA	1.0	0.7	2.3
l-leucine						
MS C18	0.9	6.9	1.0	NA	6.1	24.4
Uric acid						
MS C18	0.3	2.7	0.7	6.1	NA	8.1
l-gamma-glutamyl-l-leucine						
MS C18	0.7	5.7	2.3	24.4	8.1	NA

Abbreviations: *HSIC* Hilbert–Schmidt independence criterion, *CES-D* Center for Epidemiologic Studies-Depression Scale, *BMI* body mass index, *LSNS-6* Lubben Social Network Scale 6, *NMR* nuclear magnetic resonance spectroscopy, *MS C18* mass spectrometry in C18 mode.

No strong dependency was observed (HSIC statistics = 0.6–6.9) among the three metabolites consistently selected by HSIC Lasso (i.e., 3-hydroxyisobutyrate, gamma-linolenyl carnitine, and l-leucine). The HSIC statistics between CES-D score and these 3 metabolites (0.9–1.2) are close to those between CES-D score and sex (0.9), age (1.0), BMI (1.0), marital status (0.6), damage from the Great East Japan Earthquake (0.9), or antidepressant use (0.4) and smaller than the HSIC statistics between CES-D score and the social engagement scores LSNS-6 (1.6) and social capital score (1.4). Uric acid, a metabolite only selected by linear feature selection models, had strong dependency on sex (HSIC statistics = 29.5). l-gamma glutamyl-l-leucine, a metabolite selected only by *P* values, had strong dependency on the other selected metabolite by the same prediction model (i.e., l-leucine).

## Discussion

To the best of our knowledge, the current study utilizing 897 subjects was the largest analysis to elucidate a metabolite expression profile specific to depressive symptoms and the first study utilizing nonlinear feature selection-integrated machine learning models to predict a depressive symptoms based on metabolomic data. Our nonlinear machine learning model with feature selection successfully (i) exhibited better prediction accuracy than those achieved with frequently utilized machine learning methods, such as Lasso, SVM/KR, random forest, and partial least squares, and (ii) revealed candidate metabolite markers useful for the prediction of depressive symptoms by avoiding redundancies among metabolites as well as with covariates, including sex and BMI.

This study demonstrated that nonlinear feature selection (HSIC Lasso)-based prediction models showed better predictive powers than prediction models with assumptions of linearity (Lasso, PLS, SPLS, and multiple regression). HSIC Lasso-based models showed even better predictive power than a nonlinear prediction model (SVM/KR) with linear feature selection (Lasso or *P* < 0.05). These results suggested that feature selection without a linear assumption would improve the predictive power for depressive symptoms based on metabolome data compared with prediction models implementing feature selection with a linear assumption.

HSIC Lasso also performed better in predicting CES-D score than the most widely utilized machine learning SVM/KR (nonlinear prediction without feature selection). The prediction accuracy of SVM was lower than that of most of the other tested machine learning algorithms with feature selection, most likely due to the excessively abundant metabolite information. Whereas the previous metabolomic data-based SVM prediction of depressive symptoms utilized merely several dozen metabolite information^4,39^, our study utilized high-throughput metabolomic data, consisting of 306 metabolite markers. Han et al.^13^ comprehensively investigated the performance of SVMs to predict various diseases based on high-dimensional omics data and showed that SVM performance decreased due to overfitting, especially when a larger number of predictor variables was utilized from high-throughput omics data. Our data indicated that high-throughput data given from recent technological advances, which encompassed both useful and useless biomarkers for prediction, could have improved the prediction accuracies of models only when feature selection to extract superior biomarker sets for prediction would have been implemented. The application of high-throughput data to SVM or other algorithms without feature selection would not sufficiently improve and might even decrease the predictive power of the algorithm because random noise due to a large number of useless biomarkers would cause overfitting^13^.

Although prediction models based on metabolites and covariates increased the predictive power compared with the models based on only covariates, the degree of improvement was moderate (i.e., 0.10–0.15 in PCC and 0.05–0.15 in AUC by HSIC Lasso-based prediction models). As for a single metabolite feature, the strengths of the associations evaluated by HSIC statistics between CES-D score and the three metabolites consistently selected by HSIC Lasso (i.e., 3-hydroxyisobutyrate, gamma-linolenyl carnitine, and l-leucine) were close to those between CES-D score and marital status or the damage from the earthquake, which are reported to be one of the relatively strong environmental risk factors for depression^22,28,29^; however, the other metabolites had a smaller association with CES-D score. The proportion of CES-D score explained by metabolome datasets was significant but not as large in the current datasets, which could indicate the complexity of the depressive symptoms. In other words, a single metabolite feature (biological factor) or a single environmental factor can explain a limited proportion of the variance in the depressive symptoms. To improve the predictive power of the prediction models, feature selection models based on datasets including various biological data (not only metabolites but also genomes or brain imaging) and various environmental data could be a good approach for predicting depressive symptoms. For this multidimensional data approach, HSIC Lasso would be useful when predictor variables include quantitative variables, among which nonlinear relationships can exist.

There were only three metabolites that were consistently selected by HSIC Lasso for both quantitative and binary traits, although HSIC Lasso selected 5–15 metabolites for each prediction in the fivefold cross-validation. One of the reasons for the variety in the selected metabolites would be that HSIC Lasso uses L1 norms for penalty term, similar to classical Lasso, and selects only one strongest feature among the metabolites that are highly dependent on each other. Consequently, when several metabolites have equivalent dependency on the output variable and high dependency on each other, it can be unstable which metabolite is selected for prediction. In addition, slightly different metabolites were selected between the models to predict quantitative CES-D scores and binary CES-D traits. We performed a relatively conservative approach to find risk metabolites by focusing on only those selected by both prediction models for quantitative and binary CES-D traits.

All three metabolites consistently selected by HSIC Lasso were located on different pathways, each of which was previously suggested to be associated with depression. Three-hydroxyisobutylate is an intermediate of valine catabolism and has recently been demonstrated to act as a paracrine factor to stimulate endothelial fatty acid uptake induced by PGC-1α^40^. One of the isoforms of PGC-1α, PGC-1α1, has been suggested to modulate kynurenine metabolism and to exert a protective effect against stress-induced depression^41^. Gamma-linolenyl carnitine is one of the acylcarnitines. Decreases in a set of medium-chain acylcarnitines in patients with depression were reported in one of the largest metabolomic studies^3^. In addition, decreased plasma acylcarnitines were reported to distinguish depressed subjects from controls and were correlated with the severity of depression in both HIV-positive and HIV-negative patients^42^. l-leucine is one of the essential amino acids and was recently reported to be taken up into the brain, where astrocytes convert it to alpha-ketoisocaproate via the transamination of alpha-ketoglutarate to glutamate^43^.

We succeeded in showing several metabolites with dependency on the CES-D score in a large dataset of the Japanese population, but ethnicity needs to be considered when using the prediction model in different populations. There is accumulating evidence that ethnicity influences the metabolite profile^44^ and the biological risk markers for depression^45^. To apply the HSIC Lasso-based prediction model to a different dataset with different ethnicities, such as European and African samples, updates for the training data would be needed, i.e., using samples with ethnicities similar to those of the target population. Future machine learning studies with larger sample sizes are also recommended to cover a greater number of degrees of freedom.

The current study included several limitations. First, the outcome measure was the self-reported CES-D score, not the diagnosis of depression. The correlation between the clinician-rated severity of depressive symptoms and the CES-D score can be limited in some situations^20^. For example, the CES-D score can be high even when subjects experience appropriate reactions to a significant loss, which would not be diagnosed as depression^20^. In the current datasets, although the gap time between the CES-D measurements and the Great East Japanese Earthquake was more than two years on average, it is possible that there were subjects with high CES-D scores, but their reactions were appropriate to the significant loss resulting from the disaster. Another limitation of using the CES-D score is that the CES-D cannot rule out other diagnoses, and the high CES-D group can include a heterogeneous population in terms of these diagnoses. For example, the proportion of subjects who complained of PTSD symptoms was significantly larger in the high CES-D group. It is possible that the high CES-D group included not only subjects with only depression but also subjects with depression and PTSD (comorbidity). Second, the current study adopted a cross-sectional design. Several metabolites have dependency on the CES-D score, but we are unable to discuss the causal relationship between the CES-D score and these metabolites (i.e., whether the metabolites caused the depressive symptoms or the depressive symptoms changed the metabolite profile).

In conclusion, the current study demonstrated the usefulness of HSIC Lasso-based prediction models to analyze the metabolome datasets of depressive phenotypes because of its better predictive power than other state-of-the-art prediction models and its good interpretability in presenting a set of metabolites without redundancies or linear assumptions. Further studies should use HSIC Lasso-based prediction models with different ethnicities to investigate the generality of each risk metabolite for predicting depressive symptoms.

## Supplementary information

Supplementary Methods

Supplementary Figure S1

Supplementary Tables

## Data Availability

The datasets analyzed in the current study are not publicly available for ethical reasons but are available upon request after approval from the Ethical Committee of Tohoku University and the Materials and Information Distribution Review Committee of the Tohoku Medical Megabank Project.

## Code availability

The HSIC Lasso program code is available on the web (http://www.makotoyamada-ml.com/hsiclasso.html).
