# Supplementary material for: Improved metabolomic data-based prediction of depressive symptoms using nonlinear machine learning with feature selection
Source: Transl Psychiatry. 2020 May 19;10:157. doi: 10.1038/s41398-020-0831-9 (PMC7237664; doi:10.1038/s41398-020-0831-9)
Supplement: Supplementary file 1 — Supplementary Methods [file 41398_2020_831_MOESM1_ESM.docx]

Supplementary Methods

The Center for Epidemiologic Studies-Depression Scale (CES-D)

The CES-D was used as an indicator of depressive symptoms ^1^. The CES-D was developed to detect depressive symptoms in community populations ^1^, was validated in community populations of various ethnicities, and is one of the most popular tools used to evaluate depressive symptoms in communities ^2-6^. The respondents were asked about their depressive symptoms over the previous week through a battery of 20 questions, to which they responded by selecting “Rarely or None of the Time” (less than 1 day), “Some or a Little of the Time” (1-2 days), “Occasionally or a Moderate Amount of Time” (3-4 days), and “Most or All of the Time” (5-7 days). Among the 20 questions, 16 questions are about negative affect (e.g., “I was bothered by things that usually don’t bother me”), and these were scored at 0 (less than 1 day), 1 (1-2 days), 2 (3-4 days), and 3 (5-7 days). On the other hand, the other 4 questions assess (the absence of) positive affect (e.g., “I felt hopeful about the future), and these are reversely scored at 3 (less than 1 day). 2 (1-2 days), 1 (3-4 days), and 0 (5-7 days). The total scores ranged from 0 to 60, with a higher score suggesting severe depressive symptoms ^1^.

Although some of the questions were reversely scored, there were a certain number of subjects who answered all the questions “Rarely or None of the Time” or “Most or All of the Time”, which would be illogical. In that case, it is possible that the subjects were not paying attention to the contents of each question, and they were excluded as “unreliable answers” to increase the validity of the CES-D score, in accordance with previous studies ^7-9^.

As outcome measures, not only quantitative CES-D scores but also binary CES-D traits using cutoffs were utilized. The optimal cut off was suggested to be 16, with scores greater than or equal to this value indicating a potential depressive group, when CES-D was first developed^1, 10, 11^. When the Japanese version of the CES-D was validated for the first time, the optimal cutoff was also reported to be 16 (sensitivity 88.2%, specificity 84.8%)^8^. The correlation between CES-D score and the clinician-rated Hamilton Depression Rating Scale (HAM-D) score was 0.846 in the Japanese population^8^. Recently, another cutoff of CES-D scores (i.e., greater than or equal to 19 for depression) was also reported to be optimal for the Japanese working population (sensitivity 92.7%, specificity 91.8%)^7^. We performed prediction analyses for binary CES-D traits using both cutoff values (16 and 19) to separate the high CES-D group from the low CES-D group.

Nuclear magnetic resonance (NMR) measurements and mass spectrometry (MS) measurements

NMR and MS measurements used to develop the jMorp database are detailed in elsewhere ^12-15^. Blood samples were collected from 9:00 ~ 15:30 following a fasting period (>10 hours after the last meal) using Venoject II® tubes containing EDTA- 2Na (Terumo Corporation, Tokyo, Japan). After collection, the sample tubes were immediately inverted 10 times and stored at 4°C. These sample tubes were transported to the Tohoku Medical Megabank biobank laboratory using refrigerated containers with temperature data loggers. The total transport time was within 8 hours for most samples. The transported tubes were centrifuged at 2,330 × g for 10 min at 4°C. The plasma fraction was transferred to a liquid handling machine (Freedom EVO®, Tecan, Männedorf, Switzerland) and dispensed into MATRIX® 1.0-ml 2D barcoded screw tubes (Thermo Scientific, Waltham, MA, USA). The number of dispensed tubes was basically four per blood sample, and the plasma volume in each tube was approximately 700 μl. These samples were stored at −80°C. Samples used in the current analysis were stored at −80°C for no more than 3 years. Metabolites were extracted using a standard methanol extraction procedure. All NMR experiments were performed at 298 K using a Bruker Advance 600 MHz spectrometer (Bruker BioSpin, Billerica, MA, USA). After standard 1D nuclear Overhauser effect spectroscopy (NOESY) and Carr-Purcell-Meiboom-Gill (CPMG) spectra were measured for each sample, data were processed utilizing the Chenomx NMR Suite (Chenomx, Edmonton, Canada). Identification and quantification of metabolites were performed using the target profiling approach implemented in the Chenomx Profiler module.

Ultrahigh-performance liquid chromatography quadrupole time-of-flight MS analysis was performed on an ACQUITY Ultra Performance liquid chromatography I-class system (Waters Corp., Milford, MA, USA), which was interfaced with a Waters Synapt G2-Si quadrupole time-of-flight MS with an electrospray ionization (ESI) system utilized in positive ion mode. A C18 column (ACQUITY HSS T3, Waters Corp.) was used for liquid chromatography separation. The data collection was performed using MassLynx, v4.1 software (Waters Corp.). A NANOSPACE SI-2 HPLC (Shiseido, Tokyo, Japan) and a Q Exactive Orbitrap MS (Thermo Fisher Scientific, Waltham, MA, USA) equipped with a heated-ESI-II source were integrated into the liquid chromatography Fourier Transform MS system for negative ion mode. A HILIC column (ZIC-pHILIC, SeQuant, Darmstadt, Germany) was used for liquid chromatography separation. The data collection was performed using Xcalibur v4.1 software (Thermo Fisher Scientific). Finally, 306 metabolite features (the concentration distributions of 37 metabolites identified by NMR measurements and the distributions of the peak intensities of 269 characterized metabolites from the MS measurements) were utilized as predictive variables for the following analyses. Most of the 306 metabolite features were included among the family of organic acids and derivatives (approximately 30%), lipids and lipid-like molecules (approximately 30%), organoheterocyclic compounds (approximately 20%), and organic nitrogen compounds, phenylpropanoids and polyketides (approximately 10%), according to the classification of the Human Metabolome Database (HMDB) ^16^. The metabolites in the previously suggested pathways associated with depression (e.g., kynurenine metabolism^17^ or acyl carnitine family^18^) were also included.

Reference

1. Radloff L. The CES-D scale: A self-report depression scale for research in the general population. *Applied psychological measurement* 1977; **1**(3)**:** 385-401.

2. Furukawa T, Hirai T, Kitamura T, Takahashi K. Application of the Center for Epidemiologic Studies Depression Scale among first-visit psychiatric patients: a new approach to improve its performance. *Journal of affective disorders* 1997; **46**(1)**:** 1-13.

3. Barlow A*, et al*. Paraprofessional-delivered home-visiting intervention for American Indian teen mothers and children: 3-year outcomes from a randomized controlled trial. *The American journal of psychiatry* 2015; **172**(2)**:** 154-162.

4. Verhoeven JE*, et al*. Depression, telomeres and mitochondrial DNA: between- and within-person associations from a 10-year longitudinal study. *Mol Psychiatry* 2018; **23**(4)**:** 850-857.

5. Fancourt D, Tymoszuk U. Cultural engagement and incident depression in older adults: evidence from the English Longitudinal Study of Ageing. *Br J Psychiatry* 2019; **214**(4)**:** 225-229.

6. Zhang W*, et al*. Comparing genetic variants detected in the 1000 genomes project with SNPs determined by the International HapMap Consortium. *Journal of genetics* 2015; **94**(4)**:** 731-740.

7. Wada K*, et al*. Validity of the Center for Epidemiologic Studies Depression Scale as a screening instrument of major depressive disorder among Japanese workers. *American journal of industrial medicine* 2007; **50**(1)**:** 8-12.

8. Shima S, Shikano T, Kitamura T, Asai M. New Self-Rating Scales for Depression [Atarashii yokuutsuseijiko hyouka syakudo ni tuite]. *Clin Psychiatry* 1985; **27:** 717-723.

9. Kerr DCR*, et al*. Associations between vitamin D levels and depressive symptoms in healthy young adult women. *Psychiatry Research* 2015; **227**(1)**:** 46-51.

10. Comstock GW, Helsing KJ. Symptoms of depression in two communities. *Psychological medicine* 1977; **6**(4)**:** 551-563.

11. Weissman MM, Locke BZ. Comparison of a Self-Report Symptom Rating Scale (Ces-D) with Standardized Depression Rating Scales in Psychiatric Populations. *American Journal of Epidemiology* 1975; **102**(5)**:** 430-431.

12. Koshiba S*, et al*. Omics research project on prospective cohort studies from the Tohoku Medical Megabank Project. *Genes to cells : devoted to molecular & cellular mechanisms* 2018.

13. Tadaka S*, et al*. jMorp: Japanese Multi Omics Reference Panel. *Nucleic acids research* 2017; **46**(D1)**:** D551-D557.

14. Koshiba S*, et al*. The structural origin of metabolic quantitative diversity. *Scientific reports* 2016; **6:** 31463.

15. Saigusa D*, et al*. Establishment of protocols for global metabolomics by LC-MS for biomarker discovery. *PloS one* 2016; **11**(8)**:** e0160555.

16. Wishart DS*, et al*. HMDB 4.0: the human metabolome database for 2018. *Nucleic acids research* 2017; **46**(D1)**:** D608-D617.

17. Agudelo LZ*, et al*. Skeletal muscle PGC-1alpha1 modulates kynurenine metabolism and mediates resilience to stress-induced depression. *Cell* 2014; **159**(1)**:** 33-45.

18. Liu X*, et al*. Discovery and validation of plasma biomarkers for major depressive disorder classification based on liquid chromatography-mass spectrometry. *J Proteome Res* 2015; **14**(5)**:** 2322-2330.
